# Patterns of brain activity distinguishing *free* and *forced* actions: contribution from sensory cortices

**DOI:** 10.3389/fnint.2012.00084

**Published:** 2012-09-27

**Authors:** Wojciech Kostelecki, Ye Mei, Luis Garcia Dominguez, José L. Pérez Velázquez

**Affiliations:** ^1^Neuroscience and Mental Health Program, Hospital for Sick ChildrenToronto, ON, Canada; ^2^Institute of Medical Science, University of TorontoToronto, ON, Canada; ^3^Division of Neurology, Department of Paediatrics, Centre for Brain and Behaviour, Hospital for Sick ChildrenToronto, ON, Canada

**Keywords:** decision-making, free will, single trial classification, granger causality, magnetoencephalography (MEG)

## Abstract

The neural basis of decision-making is extremely complex due to the large number of factors that contribute to the outcome of even the most basic actions as well as the range of appropriate responses within many behavioral contexts. To better understand the neural processes underlying basic forms of decision-making, this study utilized an experiment that required a choice about whether to press a button with the right or left hand. These instances of decision-making were compared to identical button presses that were experimentally specified rather than selected by the subject. Magnetoencephalography (MEG) was used to record neural activity during these—what are being termed—*free* and *forced* actions and differences in the MEG signal between these two conditions were attributed to the distinct forms of neural activity required to carry out the two types of actions. To produce instances of *free* and *forced* behavior, cued button-pressing experiments were performed that use visual, aural, and memorized cues to instruct experimental subjects of the expected outcome of individual trials. Classification analysis of the trials revealed that cortical regions that allowed for the most accurate classification of *free* and *forced* actions primarily handle sensory input for the modality used to cue the trials: occipital cortex for visually cued trials, temporal cortex for aurally cued trials, and minor non-localized differences in MEG activity for trials initiated from memory. The differential roles of visual and auditory sensory cortices during *free* and *forced* actions provided insight into the neural processing steps that were engaged to initiate cued actions. Specifically, it suggested that detectable differences exist in the activity of sensory cortices and their target sites when subjects performed *free* and *forced* actions in response to sensory cues.

## Introduction

The complex sequence of neurophysiological events that accompanies decision-making is becoming an increasingly studied topic in neuroscience and its related fields (Hallett, 2007; Haggard, 2008; Schall, 2001). Various neuroimaging methodologies are being utilized to better understand the differences in brain activity that accompanies *free* and *forced* actions in humans. In this study, subjects undergo magnetoencephalography (MEG) neuroimaging while performing tasks that create opportunities for multiple equally appropriate courses of action as well as tasks that specifically instruct the execution of certain actions. These two types of actions (*free* and *forced*, respectively) are contrasted to determine the primary differences in MEG activity between cued actions that require a decision and those that do not.

Foundational research has revealed that brain structures involved in producing various types of *free* actions are highly dependent on the specific types of decisions being made and the experimental procedures used to elicit them. However, evidence suggests that a few key brain regions are more prominently involved in the distinct aspects of neural decision-making; particularly, the prefrontal cortex, parietal cortex, and supplementary motor area (SMA). Prefrontal cortex is commonly involved in early stages of unconscious action selection (Soon et al., 2008) and storing intended actions for delayed initiation (Haynes et al., 2007; Koechlin and Hyafil, 2007). The SMA is involved in late stages of action selection and initiation, feeling the urge to perform an action, and along with the rostral cingulate zone, in the internal generation of movements (Deiber et al., 1991; Jenkins et al., 2000; Mueller et al., 2007). Parietal regions are involved in associating perceptions with actions (Verleger et al., 2005; Keller et al., 2006) and experiencing ownership of decisions (Hallett, 2007). There are also studies that report the influence of an assortment of these regions in the neural processes generating specific types of *free* behaviors (Deiber et al., 1996, 1999). Depending on the type of decision being made, neural processes underlying decision-making can be engaged in many or all of these neuroanatomical structures. Furthermore, depending on the imaging methodology and analysis being used, different aspects of decision-making processes can be uncovered which provide insight into various neural dependencies that exist, their neuroanatomical basis and the temporal extent over which they occur.

Research has not previously focused on the sensory cortices as regions of major contribution to *free* or *forced* behaviors but it is clear, especially for actions initiated by sensory cues, that a range of experiments, neuroimaging technologies, and analysis methods must be considered when studying the neural processes occurring in these regions during *free* and *forced* actions. In this study, we investigate decision-making by examining the differences in MEG activity in subjects performing three types of button-pressing tasks that elicit *free* and *forced* responses. These tasks required the subjects to exercise a choice by pushing either a left or right button (referred to as a *free* button press) or follow a specific instruction to press a designated button (referred to as a *forced* button press). *Free* and *forced* button presses were overtly identical but differed experimentally in the stimuli used to cue each type of button press, and therefore, physiologically in the neural mechanisms transforming specific stimuli into their designated responses based on whether exact instructions needed to be followed or a decision was required for the execution of the action. In an initial experiment, subjects were instructed to respond to visual cues (data taken from Garcia Dominguez et al., 2011). In two follow-up experiments, subjects were either instructed with aural cues or required to press buttons according to memorized instructions with no immediate cue indicating the type of button press to be performed. For the visually and aurally cued experiments, the sensory cortices corresponding to the modality used to cue the trials and output from these sensory cortices were shown to be involved distinctly in the execution of *free* and *forced* button presses and were the most significant contributors to successful classification of trial type.

## Materials and methods

### MEG data acquisition

This section describes the collection of MEG data for the aurally cued and memorized trial type button-pressing experiments which differ in several details from the data collection of the visually cued experiment taken from a previous study (see Garcia Dominguez et al., 2011 for details). Both experiments were undertaken with the understanding and written consent of each subject according to the protocol required by the Hospital for Sick Children Review Ethics Board. For the aurally cued and memorized trial type experiment, MEG recordings were performed using a whole head 151 channel CTF MEG recording system with a sampling rate of 600 Hz. Head positions were continuously monitored and recordings were discarded if movements of more than 5 mm occurred within a session.

Subjects had one button placed on each side of their body and were asked to be ready to press these buttons with the index finger of either hand in response to cues. Subjects completed the required task over three epochs of 10 min, spaced by 1–2 min rest periods. Each epoch contained repeated stretches of 14 button presses with pauses in between each stretch during which the subject had to initiate the next set of 14 button presses by indicating their readiness with a right-handed button press. Within stretches, each cue was presented 0.5 s after the previous button press. Upon partitioning the data into trials, the first and last button presses of each stretch were discarded so that each trial in the analysis was nested between two other button presses.

### Aurally cued experiment

MEG recordings were performed with subjects wearing ear pieces that supplied recordings of spoken instructions for the types of button presses to perform. The instructions consisted of the words, “left,” “right,” and “free,” to indicate whether the subject should press the left, or right button, or a button of their choice, respectively. The three spoken cues were presented in a random order with presentation rates of 0.25, 0.25, and 0.5, respectively. For the “free” cue, subjects were instructed, in addition to making a decision about what button to press, to also be unpredictable about which button they pressed.

In total, seven right-handed male participants were tested, with subject ages ranging from 22 to 47 with a median age of 34. The average response times (measured from the start of the aural cue) varied between 628 and 1393 ms with a mean across subjects of 976 ms. Additional statistics about the collected dataset are provided in Table 1.

**Table 1:** **Classification analysis statistics for visually cued, aurally cued, and memorized trial type experiments**.

	**Visual cues**	**Aural cues**	**Memorized trial type**
Number of subjects	6	7	7
Mean number of trials per subject	476	197	538
Mean peak amplitude-based classification rate	0.819	0.914	0.531
95% confidence interval	0.786–0.850	0.873–0.943	0.495–0.567
*p*-value	0.021^*^	0.025^*^	0.40
GC classification rate	0.792	0.605	0.580
95% confidence interval	0.756–0.824	0.545–0.663	0.544–0.616
*p*-value	3.4 × 10^−9*^	0.0026^*^	0.0056^*^

Mean number of trials indicate the combined number of free and forced trials for all folds. The p-values for Fisher discrimination are calculated at the peak mean classification rate and compared to a chance rate of 0.5 using t-tests. Asterisks here denote significance when controlling for a false discovery rate of α = 0.05 across classification accuracies calculated for every investigated point in time. The p-values for GC classification are given for final validation classification rates compared to a chance rate of 0.5 using t-tests with asterisks denoting significance. Classification rate confidence intervals are calculated for counts aggregated across subjects and by using the Clopper-Pearson method for binomial distributions.

### Memorized trial type experiment

Subjects were required to perform *forced* and *free* button presses during prespecified and alternating stretches of 14 button presses while only receiving timing cues from a display screen. For the *forced* stretch—indicated by the displayed word “forced” at the beginning of the stretch—the subject was required to press the buttons in the order of the memorized sequence L, L, R, R, L, L, R, R, L, L, R, R, L, L where “L” and “R” indicate left and right button presses. For the *free* stretch—indicated by the displayed word “free” at the beginning of the stretch—the subject was required to press buttons of their own choosing for the duration of the 14 button press sequence and to make the sequence of button presses unpredictable. Because the instruction “free” and “forced” come at the beginning of the stretch of responses, the experiment will be described by the term “memorized.”

In total, seven right-handed participants (five male and two female) were tested, with subject ages ranging from 25 to 49 with a median age of 38. Additional statistics about the collected dataset are provided in Table 1.

### Control experiments

Control experiments were performed with 4 subjects using similar protocols to that described by Garcia Dominguez et al. (2011) for the visually cued experiment and above for the aurally cued experiment. However, subjects were asked only to attend to differences in cues without responding with a button press. Since trials were no longer initiated by the subject, cues were presented at a rate of 1 per second and data was aligned at the time of cue onset.

### General MEG preprocessing procedure

For each subject, trials with incorrect responses were discarded but no additional rejection of trials was performed.

*Free* button presses were analyzed to ensure that subjects did not overtly violate the requirement to produce unpredictable decisions. The total number of left and right-handed responses was counted to ensure that one response was not favored over the other. Additionally, conditional probabilities of left- or right-handed responses were made given the previous response were calculated to ensure local unpredictability in responses.

For both types of classification analyses that were performed, the number of *forced* left, *forced* right, *free* left, and *free* right trials were balanced in training and testing sets and excess trials were discarded. The mean number of trials across subjects that were available for the classification analyses is shown in Table 1.

### Fisher discrimination analysis

**Figure 1:**
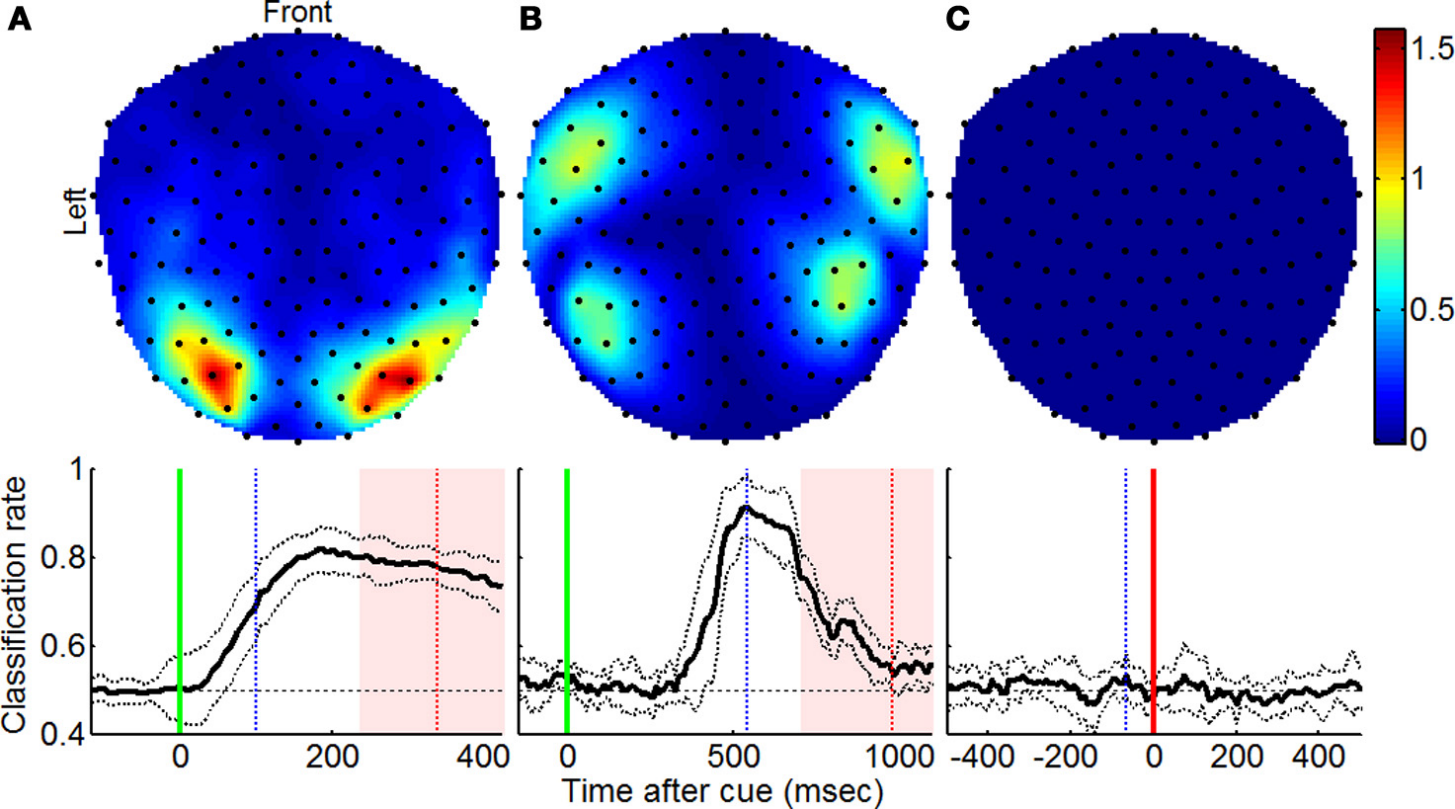
**Regions contributing to increased classification accuracy using Fisher discriminant analysis.** Top row shows topographic plots indicating sensors at which amplitude-based signal classification was most notable as described by Equation (1) for **(A)** visually and **(B)** aurally cued experiments and the **(C)** memorized instruction experiment. Bottom row shows mean classification rate across subjects over the course of the trial with vertical green, blue, and red lines denoting the time of cue onset, time for which top row plots were generated, and mean reaction times (±1 SD), respectively, and solid vertical lines indicating the point in time at which trials were aligned.

Fisher's linear discriminant analysis based on MEG signal amplitudes was performed on *forced* and *free* trials using the method described by Garcia Dominguez et al. (2011) [based on analysis methods described by Müller et al. (2004)]. This type of classification was applied independently at every point in time to show instantaneous changes in neural activity that distinguish *forced* and *free* behaviors (displayed in the bottom row of Figure 1). Additionally, the Fisher separation criterion was evaluated at individual sensors at the point of peak classification using the equation

(1) $$F\text{=}\frac{{\left(\mu_{free}-\mu_{forced}\right)}^{\text{2}}}{\sigma_{free}^{\text{2}}+\sigma_{forced}^{\text{2}}}$$

where μ_*free*_ and μ_*forced*_ are mean MEG signal amplitudes for *free* and *forced* trials at a particular time and σ^2^_free_ and σ^2^_forced_ are signal standard deviations at that particular time (displayed in the top row of Figure 1).

### Granger causality (GC) classification analysis

Selection of suitable parameter settings for the autoregressive (AR) model order, MEG signal downsampling, data window start and end times, and the extent of artifact deletion was performed according to the guidelines provided by Kostelecki et al. (2011). Settings for parameters generally agreed between experiments and across subjects so for all analyses, an AR model order of 4 was used, artifact deletion was performed by deleting the 13 principal components with highest variance, and signals were downsampled to ~45 Hz (downsampling by a factor of 14 for the visually cued experiment data which was originally sampled at 625 Hz and by a factor of 13 for the aurally cued and memorized trial type experiment data which was originally sampled at 600 Hz). For the visually cued experiment, classification was performed on cue-locked trials starting 50 ms after cue presentation and ending 100 ms before the mean reaction time. For the aurally cued experiment, trials started 100 ms after cue presentation and ended 100 ms before the mean reaction time. For the memorized trial type experiment, trials were response-locked and started 400 ms before the response and ended 100 ms before the response.

GC features were calculated by first estimating condition-specific (i.e., separately for *free* and *forced* conditions) bivariate AR models between all combinations of sensors and univariate AR models for all sensors. The resulting AR models were used to calculate GC features according to the equation

$$\varphi_{j\to i}=F_{j\to i|free}-F_{j\to i|forced}$$

where

$$F_{j\to i}=\log\frac{\sigma_{i\text{|}i}^{\text{2}}}{\sigma_{i\text{|}ij}^{\text{2}}}$$

is the GC metric evaluated from the univariate and bivariate AR prediction error variances (σ^2^_*i*|*i*_ and σ^2^_*i*|*ij*_ respectively) when the AR models estimated from training data specified with the subscript *free* or *forced* was used to calculate prediction error. Features were classified with naïve Bayes classification using 512 of the most distinct features as identified by the training set *t*-statistic comparing features from the *free* condition with those from the *forced* condition. Trials were divided into 5 balanced folds and the settings for the parameters discussed above were calculated with repeated cross-validation using the first four folds. The final classification accuracies were subsequently determined by classifying trials in the fifth withheld fold.

### GC feature analysis

**Figure 2:**
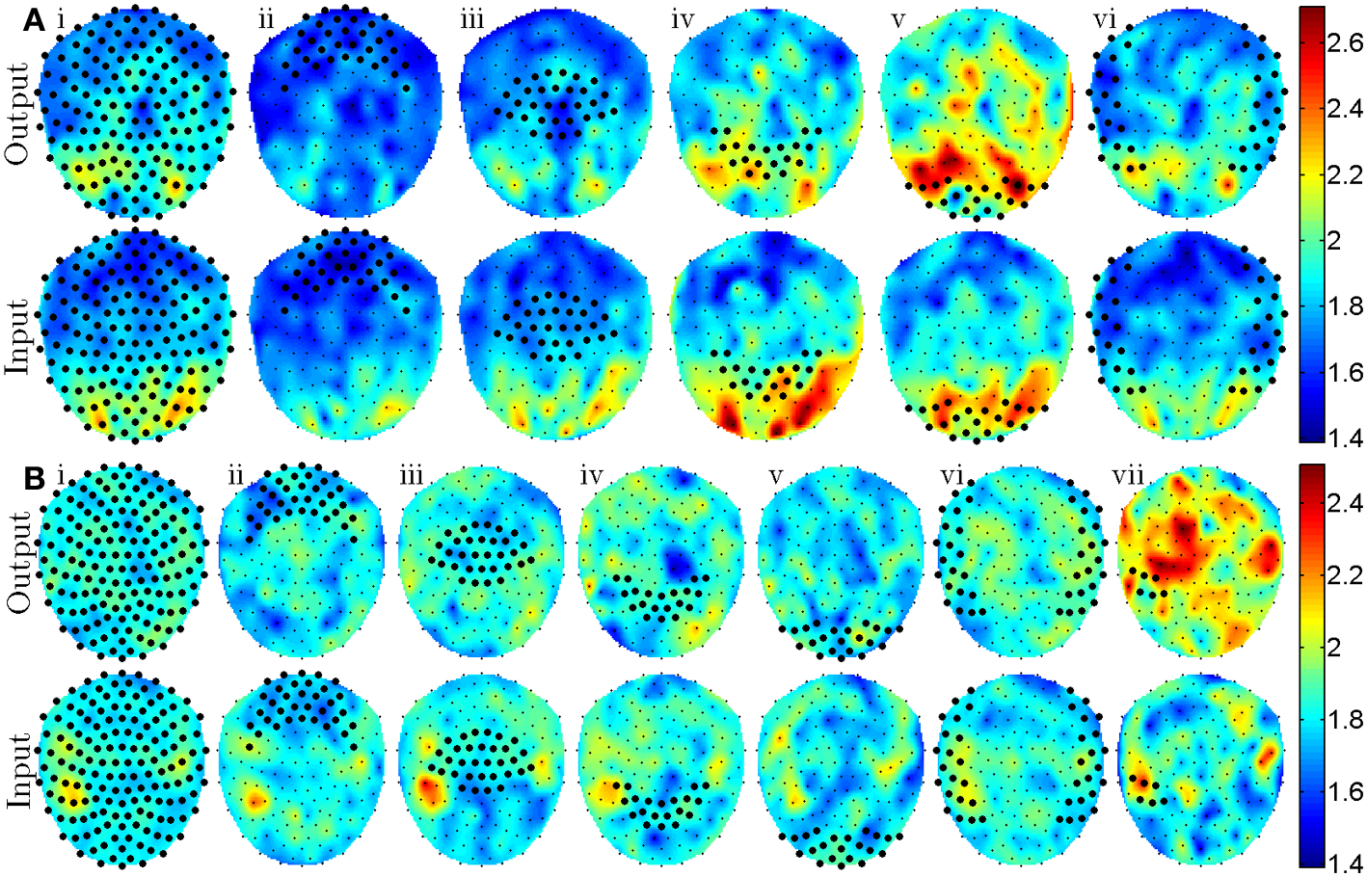
**Topographic plots show the sensor positions where the most notable differences in GC features exist [mean *t*-statistics defined by Equations (2) and (3)] for the visually cued experiment (A) and the aurally cued experiment (B).** Top rows of **(A** and **B)** indicate regions of major difference in *free* and *forced* GC features that have outputs from sensors denoted with large markers. Bottom rows of **(A** and **B)** indicate regions of major difference in *free* and *forced* GC features that have inputs to sensors denoted with large markers. Roman numerals **i–vi** denote sensor groupings that include all, frontal, central, parietal, occipital, and temporal sensors with a *post-hoc* addition of grouping **vii** that includes a portion of left temporal sensors.

The criteria for ranking the classification features—the *t*-statistic, *t*_*j*→*i*_, comparing the GC feature distributions for *free* and *forced* conditions—were averaged over spatial regions of sensors, ***Z***, and the results were plotted in Figure 2. This averaging was performed according to the equation

(2) $$c_{Z\to i}=\frac{1}{N_{Z\backslash i}}\sum_{j\in Z\backslash i}t_{j\to i}^{*}$$

to determine the extent to which the GC relationships from sensors *j* ∈ ***Z***\*i* to sensor *i* differ between the *free* and *forced* conditions. The resulting values for *c*_***Z***→*i*_ are plotted in the top rows of Figures 2A and 2B for all *i*. For all plots in Figure 2, the channel indices in ***Z*** were selected using the frontal (2A–Bii), central (2A–Biii), parietal (2A–Biv), occipital (2A–Bv), and temporal (2A–Bvi) labels supplied by the CTF MEG recording system. An additional region of interest is shown in Figure 2Bvii derived from similarities between identified spatial regions in Figure 1B (top) and Figure 2Bii–iv (bottom) as well as the approximate position of known language processing cortical regions. Similarly to Equation (2), the mean input from sensor *i* to sensors *j* ∈ ***Z***\*i* was calculated using the equation

(3) $$c_{i\to Z}=\frac{1}{N_{Z\backslash i}}\sum_{j\in Z\backslash i}t_{i\to j}^{*}$$

and the results are displayed in the bottom rows of Figures 2A and 2B. Note that in Equations (2) and (3), *N*_***Z***\*i*_ is the number of elements in ***Z***\*i* and depends on the number of grouped sensors and whether the sensor of comparison, *i*, is within ***Z***. Also note that, Equations (2) and (3) only express averaging over a group of sensor pairs but for all plots in Figure 2, averaging was also performed across subjects.

## Results

With few exceptions, subjects failed to produce truly random or unpredictable responses during the *free* condition. Conditional probabilities of free choices, given the immediately preceding choice were statistically different from a chance probability of 0.5 for almost all subjects and all experiments and often exceeded 0.65 (alternately, was below 0.35 for the opposite handed response). Although there are known limitations in human capabilities of producing random sequences of decisions (Wagenaar, 1972) and subjects in this study clearly demonstrated those limitations, there was no evidence that subjects overtly violated the experimental requirements as they followed no obvious persistent pattern in producing *free* button presses.

When trials were cued visually, amplitude-based classification resulted in 81.9% accuracy and suggested that the most pronounced signal separation occurred bilaterally at occipital sensors (Figure 1A; adapted from Garcia Dominguez et al., 2011). For aurally cued trials, the same analysis resulted in 91.4% classification accuracy and uncovered the greatest amplitude separation bilaterally at temporal sensors (Figure 1B). In both cases, the groupings of sensors corresponded to cortical regions that receive sensory input for the modality used to cue the trials. The extent to which this classification was possible was not reproduced in control experiments with *p* = 0.01 and *p* = 0.006, respectively, for Wilcoxon rank sum tests comparing peak classification accuracies from experimental and control trials. For the memorized trial type experiment, the analysis revealed poor classification rates (Figure 1C) that did not exceed chance levels. Tests for significance relative to chance classification levels are summarized in Table 1.

Analysis using GC classification revealed that the spatial arrangement of sensors that contributed most to successful classification was similar but not always identical to the results obtained from the amplitude-based classification analysis. For the visually cued experiment, a classification accuracy of 79.2% was achieved and the GC features that were most distinct between *free* and *forced* trials originated from occipital areas and terminated at parietal and higher order occipital areas. For the aurally cued experiment, a statistically significant classification accuracy of 60.5% was obtained and GC features originating at left temporal sensors and terminating at left frontal, left central, and right temporal areas were most distinct between *free* and *forced* trials. Unlike the amplitude-based classification that showed a bilateral contribution from temporal cortices, the GC-based classification revealed contribution that was more lateralized with left temporal cortex exhibiting more distinct activity across *free* and *forced* trials. Although the test for determining lateralization was *post-hoc*, it is worth noting because of the unlikeliness of the results (*p* = 8.9 × 10^−13^ for Wilcoxon signed rank test comparing GC features originating from left temporal sensors against those originating from right temporal sensors). Additionally, the left temporal sensors that contributed most to amplitude-based classification did not exactly match those that contributed to GC-based classification suggesting that different aspects of the MEG signals were utilized by the two methods of analysis. The classification accuracy for the memorized trial type experiment, although statistically successful, did not exceed 60% accuracy and did not localize to any particular region of sensors so is not visualized here.

## Discussion

This study examines a commonly overlooked aspect of basic decision-making by investigating the cortical activity that precedes *free* and *forced* actions that are made in response to experimental cues. It also demonstrates the useful application of classification algorithms, feature extraction, and the use of high temporal resolution MEG imaging to obtain insight into brain mechanisms that are often hidden behind large amounts of data, noise, and complex probabilistic relationships. In performing this study, we illustrate the importance of investigating a range of brain regions with multiple analysis tools to gain a better understanding of the neural processes underlying various forms of behavior.

Many previous studies have investigated the neural activity that distinguishes different decisions and the brain regions involved in selecting different actions from a set of options (Deiber et al., 1991, 1996, 1999; Soon et al., 2008). This study, however, explored the differences between instances of decision-derived actions from identical overt actions in the absence of a decision being made. By focusing on the classification of *free* and *forced* trials irrespective of the actual decision being made, we were able to observe another important factor involved in the cued decision-making process; that there are distinct patterns of neural activity at sensory cortices preceding the initiation of cued *free* and *forced* actions at the sensory cortices corresponding to the sensory modality used to cue actions. Although the causes of these differences are not fully explored here, these observations may be explained by there being constant top–down input from higher level cortical structures to sensory regions. Once a cue is presented and reaches the sensory cortex, output can be organized directly from the sensory cortex and affect downstream processes that lead to the response. Due to the automaticity of the experiment, higher levels of integration may not be necessary in these situations to produce *free* or *forced* responses.

An obvious complication of this study is that the experiment being tested is a type where different subjects or even the same subject at different stages of the experiment can conceivably utilize multiple strategies for producing responses. Indeed, considerable variability was observed across subjects with at least one subject in each experiment having results that were not consistent with generalizations made about the entire population. As a result, the analysis can only capture the most basic features common to all or just a portion of these strategies. This, combined with the need for hundreds of trials to perform a reliable analysis, may prevent a more detailed investigation of the cortical processes being observed in this study.

Since the classification procedures used in this study attempted to distinguish *free* and *forced* trials, the analysis does not distinguish whether the identified cortical regions are more active or less active in either experimental condition; only that they be different from one situation to the other. Typically, answering these types of questions is difficult due to incomplete understanding of how indirect measurements of localized neural activity relate to the overall function of a brain. More specific to the analysis used in this study, observing differences in AR models across experimental conditions and translating those findings into metrics of causality and neurophysiologically meaningful conclusions is still a topic of investigation. In this study, it is conceivable that sensory cortices exhibited distinct task specific activity during *forced* conditions and background non-task specific activity during *free* conditions and this would allow for the classification results. If so, it is unclear whether sensory cortices play the observed role in both *free* and *forced* conditions, just one, or whether it dependents on the cuing modality. The results can only say that the neural processing steps leading to *free* and *forced* actions are handled differently by the sensory cortices used to perceive the cues.

Also of note in our findings is the relative absence of contributions from frontal areas to the ability to discriminate *free* and *forced* trials. Typically, frontal cortex is associated with functions such as storing potential choices in working memory (Frith, 2000) and remembering the history of previous responses (Hadland et al., 2001) but is also involved in attention, planning, and long term goals. These are all functions that are expected to contribute to the experiments being studied. We explain this relative absence by noting that the task being performed is fairly automatic and tends to require less than 1 second from cue presentation to response onset. Since cues are presented in a random order, the type of monitoring and planning that might be expected from frontal cortices would be identical for both *free* and *forced* trials and therefore, not distinguishable with classification analysis. Once a cue is presented, the combination of frontal top-down input to the sensory regions used to perceive the cues along with the sensory events related to cue perception may contribute to the distinguishable activity emerging from sensory regions and later contributing to execution of the response. It is, however, a surprise that there was little frontal contribution during the memorized trial type task as this variation of the experiment was designed with the hypothesis that frontal regions would be most useful for distinguishing *free* and *forced* trials. It is possible, however, that the differences between the two conditions for the memorized trial type experiment were too subtle to be resolved with the current imaging methods and analysis.

A clear difference between the two types of analyses was that the classification rates for visually and aurally cued experiments could not be achieved to the same degree that was possible with amplitude-based classification analysis. Although the visually cued classification rate was within 2% of what was achieved with amplitude-based classification analysis, classification of aurally cued trials only reached a 60% classification rate which, although statistically significant, was 30% lower than what was obtained with the amplitude-based classification procedure. Although classification of the memorized trial type data was similarly poor, it was statistically significant using GC but not for the amplitude-based classification analysis. Despite the reduced classification rate using the GC-based analysis, examination of GC features provided insight into potential causal relationships in neural activity that might be expected in cued tasks with action-based responses. For visually cued trials, the most notable differences between *free* and *forced* trials existed in GC features that originated in visual cortex and terminated in higher visual and parietal regions (Figure 2Av). For the aurally cued experiment, differences in GC features existed primarily in features originating at left temporal sensors and terminating at frontal and central sensors (Figure 2Bvii) which is anatomically consistent with output from the left early auditory and language processing regions.

### Conflict of interest statement

The authors declare that the research was conducted in the absence of any commercial or financial relationships that could be construed as a potential conflict of interest.
